# Psychological Impact of Hyperpigmentation and Acne on the Self-Esteem and Academic Performance of International Students

**DOI:** 10.7759/cureus.93899

**Published:** 2025-10-05

**Authors:** Maherin Khan, Mayren Heshmat Abdelalim Abdalla Mansour, Hansi Zhang

**Affiliations:** 1 College of Basic Medical Sciences, Jilin University, Changchun, CHN

**Keywords:** acne, beauty standards, hyperpigmentation, mental health, psychological impact, self-esteem

## Abstract

Background: Acne and hyperpigmentation are ubiquitous problems for young adults, but they are more than visible changes. They can affect confidence, reduce peer acceptance, and distract attention from academic work. This study examined their impact on international students, considering how cultural beauty standards and peer judgment may shape these experiences.

Materials and methods: A cross-sectional survey was conducted at Jilin University, enrolling 150 international students aged 18-30 years. Participants completed a questionnaire using validated scales, including the Rosenberg Self-Esteem Scale (RSES) and Likert scales, to assess anxiety symptoms, academic effects, and perceived cultural pressure. Chi-square tests and correlation analysis were used to investigate the relationships between variables.

Results: Among 150 participants, 45% reported low to very low self-esteem, and 43% experienced anxiety symptoms. Approximately 20% indicated that acne or hyperpigmentation negatively affected their academic engagement. Cultural beauty standards have a significant influence on self-perception, particularly among individuals of East Asian descent (85%) and South Asian descent (70%). A significant association was found between ethnicity and perceived beauty pressure (χ²(4) = 19.89, p < 0.001; Cramér’s V = 0.29).

Conclusions: Acne and hyperpigmentation significantly impact students’ psychological well-being and academic performance, particularly in cultural contexts with rigid beauty standards. These findings underscore the importance of providing inclusive, culturally sensitive mental health and dermatological support within academic institutions.

## Introduction

Worldwide, acne and hyperpigmentation rank among the most prevalent dermatological conditions affecting young adults, often persisting past adolescence and impacting not only physical appearance but also contributing to significant psychological distress [1-2]. While their physical impact is well recognized, the emotional and functional consequences, such as diminished self-esteem, anxiety, and reduced academic performance, remain underexplored, particularly among international populations [3].

The existing literature largely focuses on single-ethnicity samples or general populations, providing a limited perspective on how skin conditions are experienced across diverse cultural contexts [4]. Moreover, few studies have examined how the severity of acne and hyperpigmentation is associated with psychological morbidity, or how emotional resilience and peer support may influence this relationship [5]. As international student populations become increasingly diverse, understanding how cultural beauty standards and social influences shape their psychological and academic outcomes has become particularly important [6-7].

International students are uniquely positioned at the intersection of academic stress, cultural adaptation, and evolving social norms. For students with visible skin conditions, these challenges may be intensified by internalized beauty standards and perceived external judgment. However, current research rarely explores how these psychosocial factors intersect with dermatological conditions to affect students’ emotional well-being and academic engagement [8-9].

This study aims to address these gaps by examining the psychological impact of acne and hyperpigmentation on self-esteem and academic performance among international students. It further investigates the role of cultural beauty standards, social pressures, and cultural diversity in shaping these outcomes. By including participants from diverse ethnic backgrounds, including South Asian, East Asian, Middle Eastern, European, and African individuals, this research offers a culturally inclusive perspective on the connections between skin conditions, mental health, and academic performance. The findings from this study may inform culturally sensitive support strategies to improve psychological well-being and academic outcomes among affected students.

## Materials and methods

Study design and participants

This research employed an analytical cross-sectional design, incorporating both descriptive statistics and inferential analyses (chi-square and correlation tests) to assess the associations between variables. Specifically, the study aimed to explore how beauty standards and social influence contribute to these impacts, with a focus on international students. The research was conducted at Jilin University, China, from March 2025 to April 2025. Ethical approval was obtained from the Ethics Committee of the College of Basic Medical Science, Jilin University, under ethics exemption approval number (AF-BMSIRB-01-07). Participants were thoroughly informed about the study's objectives, and their consent was obtained in a proper manner. Participants were informed that anonymized quotes may be included in publications. All quotes presented have been de-identified to maintain participant confidentiality.

The study targeted 150 international university students (aged 18-30) from diverse ethnic backgrounds, including those of South Asian, East Asian, Middle Eastern, African, and European descent. A snowball sampling technique was employed, wherein initial participants were encouraged to refer others who met the inclusion criteria. To ensure relevance to the research topic, inclusion was limited to international university students who self-reported experiencing facial acne and/or skin pigmentation. This method facilitated a broader network of participants within the university community. Exclusion criteria include students with diagnosed psychiatric conditions unrelated to appearance (including post-traumatic stress disorder or other mental health disorders under treatment) and those who had experienced a recent major bereavement or traumatic event within the past three months were excluded to reduce confounding. We did not exclude students based on their academic performance (GPA), as it was assessed through self-reported items rather than transcript-based GPA. The psychological impact in this study was operationalized as a composite of self-esteem (as measured by the Rosenberg Self-Esteem Scale (RSES) scores), self-reported anxiety symptoms (captured using Likert-scale items), and self-perceived academic engagement.

To minimize duplicate submissions, the online survey could be submitted only via QR code. During data cleaning, we screened for duplicate responses by checking identical timestamps, identical demographic answers, and identical open-ended responses. Suspected duplicates were removed before final analysis, resulting in 23 responses being excluded. Additionally, responses with implausibly short completion times were excluded. The final number of valid responses included in the analysis was 150.

Severity of skin condition

The severity of acne and hyperpigmentation was assessed through self-reported categories in the questionnaire. Participants were asked to rate their skin condition as mild, moderate, or severe, based on their own perception of lesion visibility and persistence. No clinical dermatological examination was conducted; the responses reflected the participants’ subjective experiences of their skin conditions.

Rosenberg Self-Esteem Scale

This widely used scale consists of 10 items to assess participants’ overall self-esteem. The RSES provides insight into how students perceive their worth in relation to their skin concerns. Participants’ responses were scored on a four-point Likert scale, ranging from strongly agree to strongly disagree. RSES is a 10-item scale widely used to measure global self-esteem. It is available in the public domain and free to use [10].

Cultural beauty standard perception

An original set of questions was developed to assess how students from diverse cultural backgrounds perceive beauty standards related to skin conditions. The questions were adapted to capture varying attitudes toward beauty standards across ethnicities and cultures.

The questionnaire included items covering various themes, such as media portrayal of “ideal” skin, pressure from peers or family, and personal attitudes toward beauty and self-worth. A five-point Likert scale (ranging from “strongly disagree” to “strongly agree”) was used to capture participants' level of agreement with each statement [11]. This measure of cultural beauty standard perception reflects perceived social and cultural pressure from media, peers, and family regarding skin appearance.

Before finalizing, the questionnaire was reviewed by two faculty members to ensure clarity and relevance. It was then pilot-tested with a small group of students to gather feedback on the wording and flow, leading to a few minor adjustments. No copyrighted or licensed tools were used.

Academic performance

Academic performance in this study was assessed using self-reported questionnaire items covering (1) class participation, (2) ability to concentrate during lectures and study sessions, and (3) perceived impact of skin condition on assignment/presentation confidence. Each item used a five-point Likert scale (1 = strongly disagree to 5 = strongly agree). For analysis, responses indicating decreased participation or concentration were coded as indicating impaired academic engagement. We did not collect formal GPA/transcript data.

Data analysis

The data collected from the survey were analyzed using the free statistical software GNU PSPP (Free Software Foundation, Boston, MA, USA). Descriptive statistics were calculated to summarize demographic information, including age, gender, and ethnicity. Chi-square tests were used to assess the associations between categorical variables (e.g., ethnicity and perception of cultural beauty standards). Correlation analysis was performed to examine the relation between self-esteem, cultural pressure, and severity of skin conditions. Pearson correlation was used to examine relationships between continuous variables, such as Likert-scale scores on perceived social pressure and self-esteem. When variables were ordinal or non-normally distributed, such as when the severity of acne or pigmentation was reported in categories (e.g., mild, moderate, severe), the Spearman rank correlation was applied. A significance level of p < 0.05 was considered statistically significant for all tests. Potential confounders (age, gender, and ethnicity) were considered in the analysis. Where sample sizes permitted, subgroup analyses were conducted; however, multivariable regression models were not performed due to the limited sample size.

Open-ended response and qualitative summary

The survey included optional open-ended questions that invited participants to describe how their skin condition affected their daily lives. These free-text responses were analyzed using basic thematic summarization: two authors independently read the responses, conducted initial coding to identify recurring ideas, and then met to consolidate codes into themes. Discrepancies were resolved through discussion to reach a consensus. These qualitative data are presented as illustrative quotes in the Results section.

## Results

**Figure 1:**
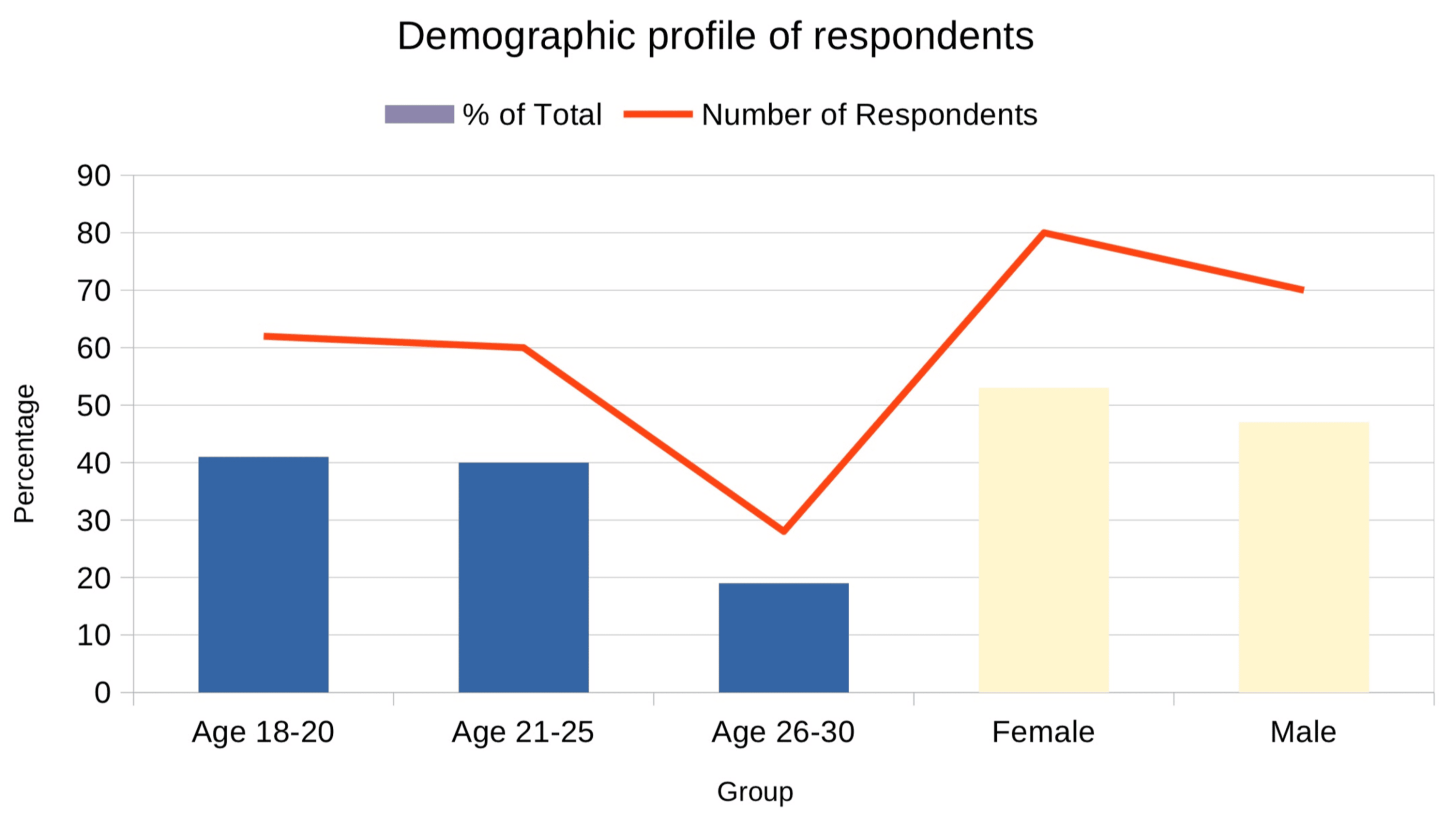
Distribution of participants by age group and gender

A total of 150 university students participated in the study. The age distribution showed a predominance of younger adults, with 41% of respondents aged 18-20 years, 40% aged 21-25 years, and the remaining 19% falling within the 26-30 years age bracket. The mean age of the participants was 21.8 ± 2.7 years (range 18-30 years). This reflects the study’s focus on a population where skin conditions, such as acne and hyperpigmentation, are particularly prevalent and have a significant psychological impact. In terms of gender, the sample consisted of 53% female and 47% male respondents, providing a balanced perspective on gender-related differences in psychological responses to dermatological concerns (Figure 1).

**Figure 2:**
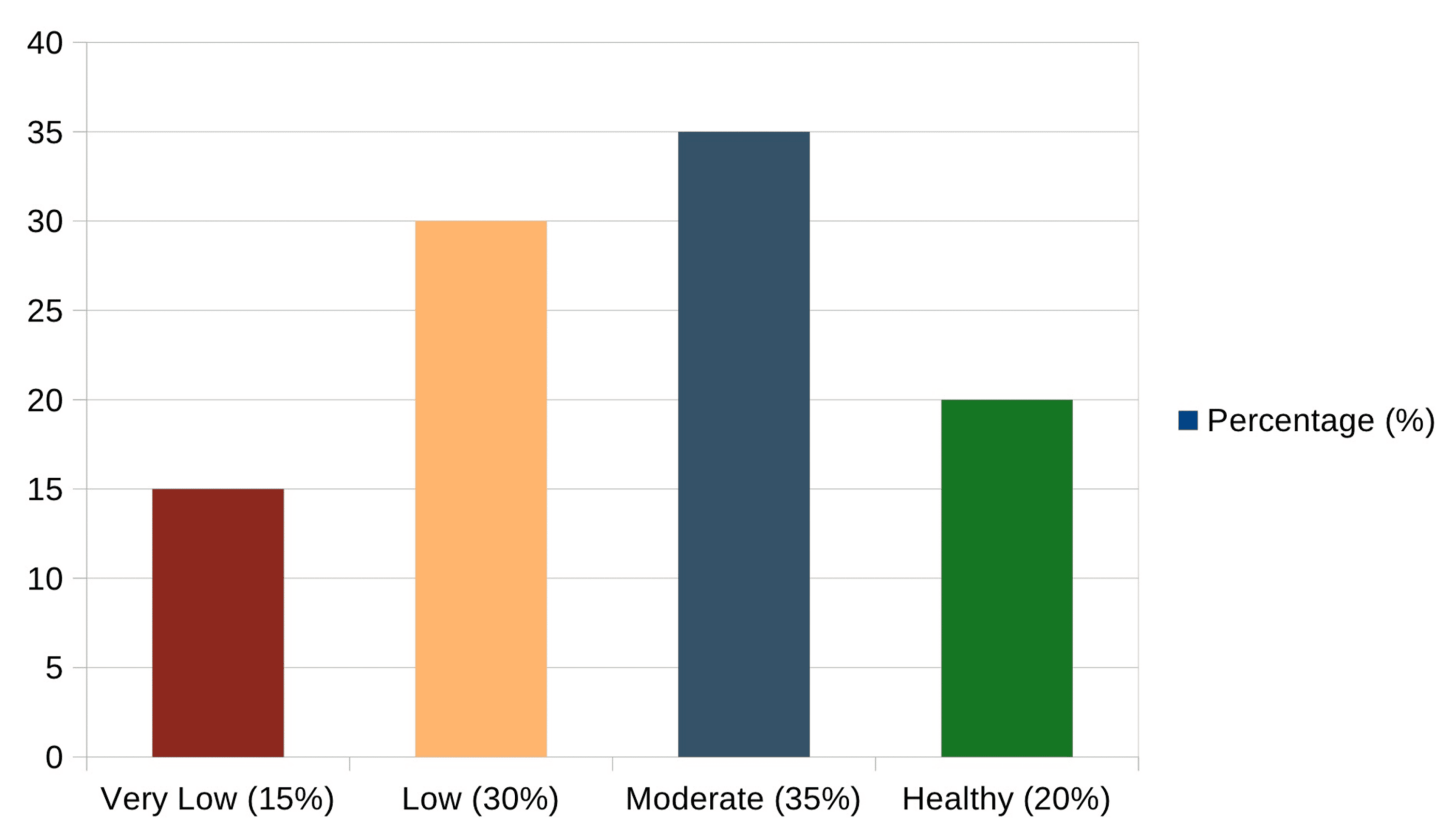
Distribution of participants’ self-esteem scores (N = 150) Measured using the Rosenberg Self-Esteem Scale (RSES; Rosenberg [10]).

RSES highlighted a clear psychological impact of acne and hyperpigmentation on participants' self-worth. About 15% of individuals fell into the very low self-esteem category, often describing persistent feelings of inadequacy and emotional distress linked to their appearance. Another 30% reported low self-esteem, with many expressing social discomfort and self-consciousness, especially in public settings. 35% of participants were found to have moderate self-esteem, indicating a mixed experience, as they felt confident at times but were still affected by breakouts or visible marks. Only 20% of respondents showed signs of healthy self-esteem, suggesting they were largely unbothered by their skin condition or had developed effective coping strategies over time (Figure 2).

Additionally, 43% of all participants reported experiencing symptoms consistent with mild to moderate anxiety, including social withdrawal, heightened self-awareness, and emotional fatigue. These psychological effects were notably more common in those with persistent facial hyperpigmentation than with transient acne alone. A narrative emerged through qualitative feedback. A 21-year-old female shared, "Almost always feel self-conscious compared to how I was a few years ago."

The psychological burden of acne and hyperpigmentation often went beyond appearance. Around 20% of participants shared that their skin condition had a noticeable impact on their academic life. Some described avoiding class participation, feeling hesitant during presentations, or struggling to concentrate, especially during flare-ups, because they felt judged or overly aware of how others might perceive them.

**Figure 3:**
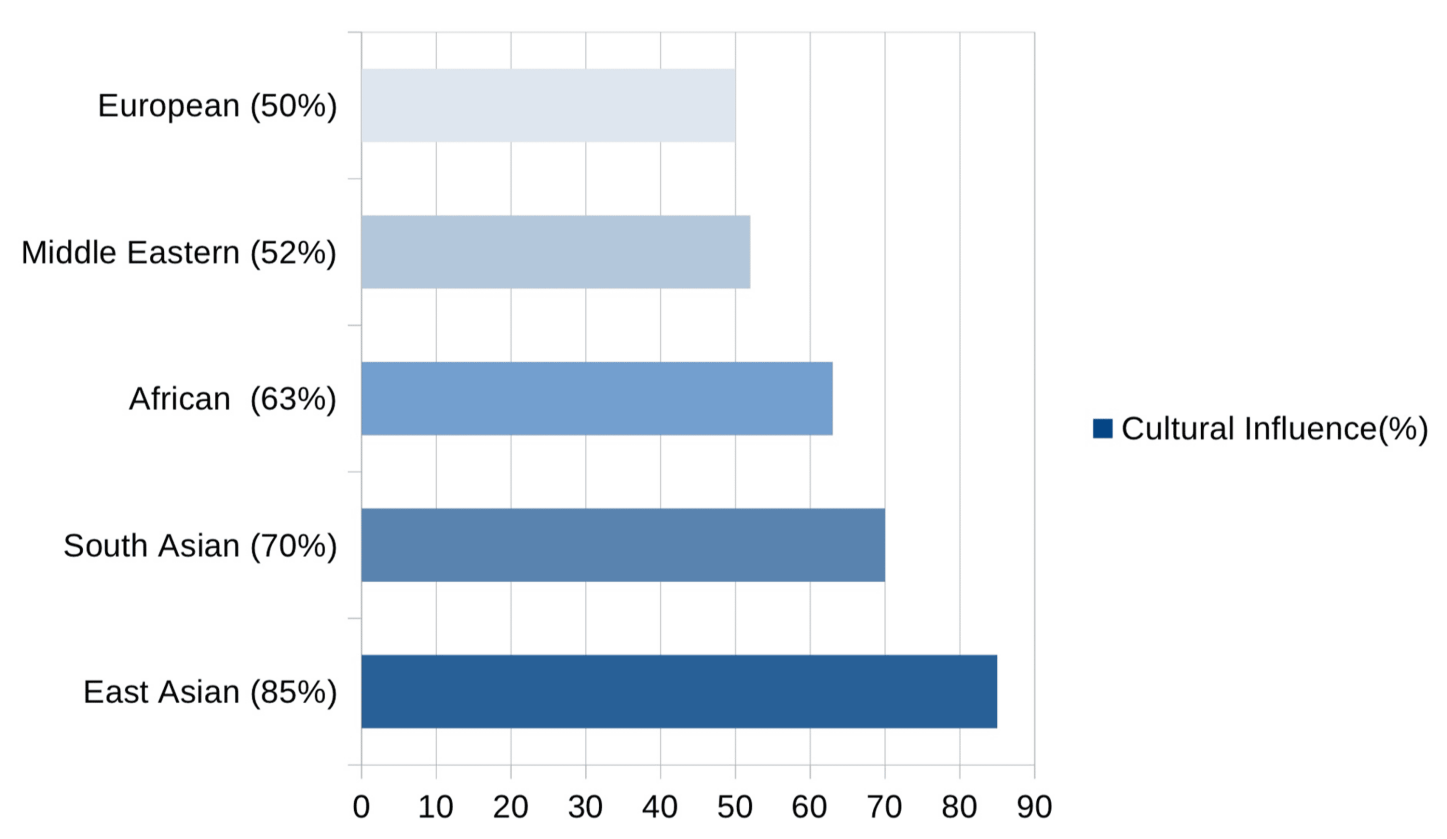
Influence of cultural beauty standards on self-perception of skin condition Categorized by ethnic group. Responses measured on a five-point Likert scale (Likert [11]).

When participants responded to the Likert-scale item asking whether their country’s beauty standards affected how they perceived their own skin, the distribution of responses varied significantly across ethnic groups (χ² = 11.25, df = 4, p = 0.024). A large majority of East Asian respondents (85%) said they felt influenced by ideals that favored clear or fair skin, often linking this pressure to feelings of inadequacy or heightened self-consciousness. Similarly, 70% of South Asians and 63% of African respondents reported being affected by cultural beauty norms. Among Middle Eastern respondents, just over half (52%) felt these standards shaped how they viewed their skin, while 50% of European respondents also acknowledged the impact of societal expectations. These numbers reflect the significant role that cultural ideals continue to play in shaping personal identity, particularly in relation to appearance-related concerns such as acne and hyperpigmentation (Figure 3).

A chi-square test of independence was conducted to assess the relationship between ethnicity and the influence of cultural beauty standards on the perception of skin conditions (Table 1).

**Table 1:** Chi-square analysis of ethnic background and perceived cultural pressure Responses measured on a five-point Likert scale (Likert [11]).

Ethnicity	Yes (influenced)	No (not influenced)	Total
East Asian	34	6	40
South Asian	21	9	30
African	19	11	30
Middle Eastern	16	14	30
European	10	10	20
Total	100	50	150
(χ²(4, N = 150) = 19.89, p < 0.001, Cramér’s V = 0.29)			

The association was statistically significant, χ²(4, N = 150) = 19.89, p < 0.001, indicating that cultural background has a significant effect on how individuals internalize societal beauty ideals. The Cramér’s V value was 0.29, reflecting a moderate effect size.

**Figure 4:**
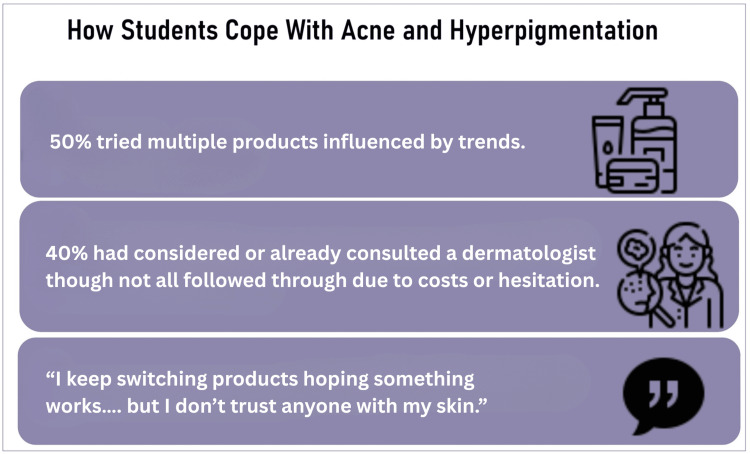
Summary of responses

Many participants turned to various coping strategies in response to their skin concerns. Over half (55%) reported trying a range of topical treatments and skincare products, frequently changing brands or routines based on social media trends or peer recommendations. Meanwhile, 40% had either consulted a dermatologist or had considered doing so. However, some individuals mentioned that financial limitations, fear of judgment, or cultural beliefs about skin health made it difficult for them to seek professional care. These patterns reflect not only a high level of concern and willingness to act but also a gap in access to reliable, structured guidance on managing acne and hyperpigmentation, particularly among young adults navigating these issues on their own (Figure 4).

## Discussion

This study reveals an extraordinary fact that is often ignored: diseases like hyperpigmentation and acne have implications extending far beyond their appearance. These effects also become entrenched in the psychological and academic lives of overseas students. In contrast to the prevailing narrative, which separates dermatological matters from mental health or learning [12], we found that students’ insecurities about their skin have a significant negative impact on their self-image, emotional resilience, and class participation. Many participants hold the view that the visibility of their skin conditions is a sign of social approval, academic worth, and even their cultural origins.

A theme that cut across many participants’ lives was the emotional suffering caused by dermatological distress within academic spaces. One student observed, “At times, my skin condition affects my self-confidence in front of others as well as my ability to focus on learning. This can be attributed largely to my psychological resistance and the inclusive attitude of people around me to all forms of physical diversity and imperfection.” This statement captures not just individual vulnerability but also highlights the environmental and cultural factors that either cushion or compound psychological strain.

When put into context with the literature, these findings are consistent with outcomes that correlate skin stigma with reduced self-esteem and depressive symptoms [13]. Our research extends the debate further by demonstrating how such psychological effects interface with academic motivation and achievement, particularly from an international perspective. In contrast to past research, which focuses on Western populations [14-15], this research puts front and center how cultural beauty standards and societal expectations of "clear skin" disproportionately affect students from non-Western backgrounds [16-18]. This is where intersectionality comes to the fore. For international students, moreover, African, South Asian, or Southeast Asian students, race, color, social displacement, and dermatological illness are mutually entwined. Having arrived in a new cultural environment where beauty norms prioritize smooth, blemish-free skin, students often experience an immediate, exaggerated sense of inadequacy. One summed it up thus: "When I looked around, it was like everyone had flawless skin. After coming to China, that feeling was even more so-I felt really insecure. I missed most classes, and my grades suffered because of it."

This evidence illustrates how internalized beauty ideals, particularly when defined by media or homogeneous local standards, have the potential to destabilize students' feelings of belonging and impact their classroom engagement. Unlike domestic students, international students must contend with academic and cultural adjustments, as well as the psychological burden of not aligning with the aesthetic ideals of their new surroundings. This combined vulnerability is commonly discounted in traditional academic support systems.

This study has several limitations. First, acne severity and hyperpigmentation were based on self-reported grades without objective clinical evaluation, which may have introduced reporting bias. Second, the cross-sectional design limits the ability to establish causal relationships between skin conditions and their psychological or academic impact. Future research should consider longitudinal designs to examine causality more conclusively, as well as intervention-based studies to evaluate the effectiveness of skin-positive campaigns, counseling, or individually tailored psychological support programs for students with dermatological disorders. Additionally, since the study employed snowball sampling at a single institution, the findings may not be fully generalizable to all international student populations.

## Conclusions

This study highlights that acne and hyperpigmentation among international students are associated with decreased self-esteem, higher anxiety, and perceived impairment in academic functioning. The research underscores the need for dermatological disorders to be regarded not only as cosmetic concerns but also as contributors to mental health and academic challenges. In this regard, academic institutions should introduce programs of awareness, counseling, and mentorship to support affected students and address skin-related stigma. Promoting skin-positive campaigns and ensuring accessible psychosocial support can reduce the psychological burden and foster a healthier, more inclusive learning environment.

These findings have direct consequences for institutional responsibility. Universities must do more than offer general counseling services. They need to foster inclusive spaces that acknowledge the psychosocial impacts of body image, race, and academic achievement. This could include skin-positive campaigns and prominence in student literature, affirmation groups, and mentorship schemes that embrace varied experiences, academic staff training to recognize and support students with psychosocial distress related to body image, and affordable dermatological care and wellness workshops integrated into university health services.
